# Gamisoyo-San Ameliorates Neuroinflammation in the Spinal Cord of hSOD1^G93A^ Transgenic Mice

**DOI:** 10.1155/2018/5897817

**Published:** 2018-06-25

**Authors:** MuDan Cai, Eun Jin Yang

**Affiliations:** Department of Clinical Research, Korea Institute of Oriental Medicine, 1672 Yuseong-daero, Yuseong-gu, Daejeon 305-811, Republic of Korea

## Abstract

Amyotrophic lateral sclerosis (ALS), a progressive disorder, causes motor neuron degeneration and neuromuscular synapse denervation. Because this is a complex disease, there are no effective drugs for the treatment of patients with ALS. For example, riluzole is used in many countries but has many side effects and only increases the lifespan of patients by approximately 2-3 months. Therefore, patients with ALS often turn to complementary and alternative medicine, such as acupuncture, homeopathy, and herbal medicine, with the hope and belief of recovery, despite the lack of definite evidence on the efficacy of these methods. Gamisoyo-San (GSS), a herbal medicine known to improve health, has been used for stress-related neuropsychological disorders, including anorexia, in Asian countries, such as China, Korea, and Japan. To evaluate the effects of GSS on the spinal cord, we investigated the expression of neuroinflammatory and metabolic proteins in symptomatic hSOD1^G93A^ mice. We observed that GSS reduces the expression of glial markers, including those for microglia and astrocytes, and prevents neuronal loss. Moreover, we found that GSS inhibits the expression of proteins related to Toll-like receptor 4 signaling and oxidative stress, known to cause neuroinflammation. Notably, GSS also regulates metabolism in the spinal cord of transgenic mice. These results suggest that GSS could be used for improving the immune system and increasing the life quality of patients with ALS.

## 1. Introduction

Amyotrophic lateral sclerosis (ALS) is a fatal neurodegenerative disease characterized by the loss of motor neurons and muscular paralysis. It is a complex syndrome causing the progressive degeneration of motor neurons in the central nervous system (CNS) and denervation of neuromuscular synapses in the peripheral nervous system. However, the cause and pathogenesis of ALS remain unclear until now, despite some studies demonstrating that excitotoxicity, oxidative stress, endoplasmic reticulum stress, and immune and inflammatory responses accompany motor neuron degeneration [1–3]. A point mutation in the gene encoding the Cu^2+^/Zn^2+^ superoxide dismutase 1 (SOD1) was shown to cause an ALS-like phenotype in mice; hence, mice harboring this mutation have been used as a model for ALS for determining ALS pathological mechanisms and developing drugs against this disease [4].

Neuroinflammation is defined as nonneuronal cell toxicity and is established as an important factor, not only in the pathogenesis of ALS but also in many other neurodegenerative diseases, including Parkinson's disease (PD), Alzheimer's disease (AD), and multiple sclerosis [5–7]. In addition, the relationship between neuroinflammation and disease progression has been demonstrated in ALS animal models [8–10]. In ALS, nonneuronal cells, including astrocytes and microglial cells, as well as peripheral immune cells, contribute to the immune response via the activation of Toll-like receptors (TLRs) in the CNS [11, 12]. Astrocytes and microglial cells expressing the mutant SOD1 (mSOD1) protein have been shown to accelerate disease progression compared with wild-type microglia and astrocytes [13, 14]. Other nonneuronal cells, such as oligodendrocytes, have also been shown to contribute to motor neuron injury, although through noninflammatory mechanisms [15, 16]. Nonneuronal cells secrete proinflammatory factors including tumor necrosis factor- (TNF-) *α*, interleukin 1a, and complement component 1q, thus contributing to neuronal cell death [17]. Oxidative stress and neuroinflammation are closely linked with regard to inducing cell death in neurodegenerative diseases, such as AD, PD, and ALS [18]. More specifically, Blasco et al. observed a correlation between several clinical parameters, including total antioxidant status, 8-hydroxy-2′-deoxyguanosine and malondialdehyde levels, and inflammation and oxidative stress markers in patients with ALS [19]. TNF-*α* contributes to neurodegeneration by promoting the formation of reactive oxidative species (ROS) and activating the nuclear factor-kappa B (NF-*κ*B) in glial cells, thus inducing neuronal cell death, which in turn activates microglia and astrocytes in the CNS.

Because of the diverse mechanisms underlying ALS, there are still no drugs for the effective treatment of patients with the disease. One of the few available drugs, riluzole, has been used in several countries for the treatment of ALS; however, it has many side effects and only prolongs the lifespan of patients by 2-3 months [20]. Therefore, complementary alternative medicines, including acupuncture, homeopathy, and herbal medicine, have been another resort for ALS patients, in their hope for recovery, despite the fact that there is no direct evidence on the efficacy of these treatments.

Gamisoyo-San (GSS), a herbal product known for its benefits in women's health, consists of Paeoniae Radix, Atractylodis Rhizoma Alba, Anemarrhenae Rhizoma, Lycii Radicis Cortex, Angelicae Gigantis Radix, Poria Sclerotium, *Liriope* Tuber, Rehmanniae Radix Crudus, Gardeniae Fructus, Phellodendri Cortex, Platycodi Radix, and Glycyrrhizae Radix et Rhizoma [21, 22]. GSS has often been used to treat symptoms of stress-related neuropsychological disorders, anorexia, and headache in menopausal women and has been reported to alleviate symptoms in women treated for breast cancer [22–26]. GSS inhibits the inducible nitric oxide synthase (iNOS), cyclooxygenase 2 (COX2), and TNF-*α* and exerts anti-inflammatory effects on macrophages [27]. Based on our observations, GSS reduces neuroinflammation by inhibiting the expression of TLR4 and cluster of differentiation (CD) molecule 11B, as well as of oxidative stress-related proteins in the gastrocnemius muscle of human SOD1 (hSOD1) transgenic (Tg) mice (Park et al., 2018, unpublished results). These mice carry a single amino acid substitution of glycine to alanine at the 93rd codon (hSOD1^G93A^) and have been used as a model of ALS. Therefore, we sought to examine the effects of GSS on neuroinflammation in the spinal cord of these mice. We found that GSS reduces the expression of microglial and astrocytic markers and prevents the loss of neurons. In addition, GSS inhibits the expression of TLR4-related signaling proteins, such as TLR4, CD14, and COX-2, and of oxidative stress-related proteins, including transferrin and heme oxygenase 1 (HO-1), thus causing neuroinflammation. Further, GSS regulates metabolism in the spinal cord of hSOD1^G93A^ Tg mice. These results suggest that GSS could be used for strengthening the immune system and improving the life quality of patients with ALS.

## 2. Materials and Methods

### 2.1. Animals

Hemizygous 5-week-old hSOD1^G93A^ mice carrying a single amino acid substitution of glycine to alanine at the 93rd codon were purchased from the Jackson Laboratory and maintained in our facility. All mice were allowed access to water and food ad libitum and were maintained under constant temperature (21 ± 3°C) and humidity (50 ± 10%) on a 12 h light/dark cycle (lights on 07:00–19:00). Offspring were genotyped by PCR, as previously described [28]. For the experiments, 2-month-old female mice were randomly divided into three groups of 4 mice each: non-Tg (Non-Tg), Tg, and Tg mice treated with GSS (Tg + GSS). Animal treatment and maintenance were performed in accordance with the animal care guidelines of the Korean Institute of Oriental Medicine, Daejeon, Korea (IACUC experiment approval number 15-036).

### 2.2. Materials

GSS was purchased from the HANKOOK SHINYAK Corporation (Chungcheongnam-do, South Korea). Primary antibodies used for Western blotting were as follows: anti-ionized calcium-binding adapter molecule 1 (Iba-1; 1 : 1000; Wako, Japan), anti-glial fibrillary acidic protein (GFAP; 1 : 3000; Millipore, MA, USA), anti-survival motor neuron (SMN; 1 : 1000; Santa Cruz Biotechnology, CA, USA), anti-TLR4 (1 : 1000; Santa Cruz Biotechnology), anti-CD14 (1 : 1000; BD Pharmingen, CA, USA), anti-COX2 (1 : 1000; Abcam, MA, USA), anti-transferrin (1 : 1000; Santa Cruz Biotechnology), anti-HO1 (1 : 1000; Abcam), anti-Bcl2 associated X (Bax, 1 : 1000; Santa Cruz Biotechnology), anti-phospho 5′-adenosine monophosphate-activated protein kinase (pAMPK; 1 : 1000; Cell Signaling, MA, USA), anti-AMPK (1 : 1000; Cell Signaling), and anti-phospho mammalian target of rapamycin (mTOR; 1 : 1000; Cell Signaling). Anti-glyceraldehyde 3-phosphate dehydrogenase (GAPDH; 1 : 1000; Santa Cruz Biotechnology) was used to control for protein loading. Peroxidase-conjugated secondary antibodies were purchased from Santa Cruz Biotechnology.

### 2.3. GSS Treatment

GSS (1 g/kg, p.o.) was administered once a day for 6 weeks, starting from 2 months of age (presymptomatic stage). As a control, the same volume of distilled water was administered orally in both the Non-Tg and the Tg groups, following the same time schedule as for GSS.

### 2.4. Western Blotting

At the day after the last administration of GSS, we sacrificed the mice and collected the spinal cord tissues. Mice were anesthetized with an intraperitoneal injection of pentobarbital (2.5 mg/g) and perfused with phosphate-buffered saline (PBS). The lumbar spinal cord (L4-L5) of each mouse was dissected and homogenized in RIPA buffer (50 mM Tris-HCl, pH 7.4; 1% NP-40; 0.1% SDS; 150 mM NaCl) containing a protease inhibitor cocktail (Calbiochem, CA, USA). Homogenized tissues were centrifuged at 14,000 rpm for 15 min at 4°C, and the supernatants were kept for further analysis. The protein concentration was determined using the BCA assay kit (Pierce, IL, USA). The samples were denatured with sodium dodecyl sulfate sampling buffer, separated through SDS-PAGE electrophoresis, and transferred to a PVDF membrane (Bio-Rad, CA, USA). For the detection of target proteins, membranes were blocked with 5% nonfat milk in TBS (50 mM Tris-HCl, pH 7.6, 150 mM NaCl), incubated first with the various primary antibodies and then with horseradish peroxidase-conjugated secondary antibodies, and finally visualized using the SuperSignal West Femto Substrate Maximum Sensitivity Substrate (Thermo Fisher Scientific, WI, USA). The ChemiDoc imaging system was used to detect the immunoreactive bands (Bio-Rad, CA, USA), which were then quantified by using the NIH ImageJ program.

### 2.5. Statistical Analysis

All data were analyzed using GraphPad Prism 5.0 (GraphPad Software, CA, USA) and presented as the mean ± standard error of the mean (SEM). Western blot results were analyzed using one-way analysis of variance (ANOVA), followed by Newman-Keuls' *post hoc* tests for multiple comparisons. Statistical significance was set at *p* < 0.05.

## 3. Results

### 3.1. GSS Attenuates Neuronal Loss in the Spinal Cord of hSOD1^G93A^ Tg Mice

**Figure 1:**
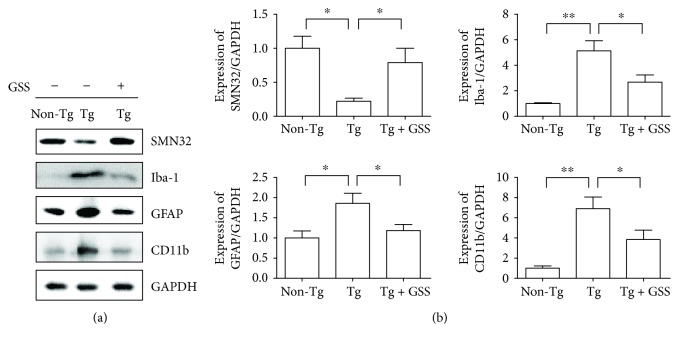
GSS administration increases motor neuron survival and modulates inflammation in the spinal cord of hSOD1^G93A^ transgenic (Tg) mice. (a) Representative Western blot showing the expression of SMN (marker of motor neurons), Iba-1 or CD11b (microglia activation markers), and GFAP (astrocyte activation marker) in the spinal cord of control (Non-Tg), hSOD1^G93A^ Tg, and Tg mice treated with GSS (Tg + GSS). GAPDH was used as a loading control. (b) Quantitative analysis of the normalized levels of SMN, Iba-1, GFAP, and CD11b against GAPDH. Data represent the means ± SEM (*n* = 4) and were evaluated by one-way ANOVA and Newman-Keuls' *post hoc* test. ^∗^*p* < 0.05 and ^∗∗^*p* < 0.01.

To determine whether GSS administration affects neuronal cell death and neuroinflammation, we collected the L4-L5 spinal cord region of Non-Tg, Tg, and Tg + GSS mice and performed Western blot analysis for various markers. The SMN protein is ubiquitously expressed throughout the body but is enriched in spinal cord motor neurons [29, 30]. We found that the expression of SMN32 was significantly lower (4.5-fold) in Tg than in Non-Tg mice, while treatment of Tg mice with GSS significantly attenuated neuronal loss by 3.6-fold (Figure 1). To examine the effects of GSS treatment on microglial and astrocytic activation in the spinal cord of hSOD1^G93A^ Tg mice, we analyzed the expression of Iba-1, CD11b, and GFAP by Western blotting. As shown in Figure 1, the levels of Iba-1 and GFAP were significantly lower (1.9- and 1.6-fold, resp.) in the spinal cord of Tg mice treated with GSS than in untreated Tg mice.

### 3.2. GSS Inhibits TLR4 Signaling-Related Proteins in the Spinal Cord of hSOD1^G93A^ Tg Mice

**Figure 2:**
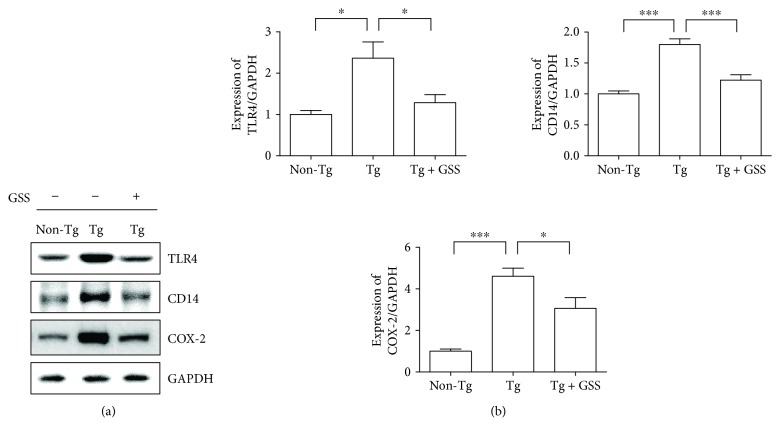
GSS administration downregulates TLR4 signaling-related proteins in the spinal cord of hSOD1^G93A^ transgenic mice. (a) Representative Western blot images displaying the protein levels of TLR4, CD14, and COX2 in the spinal cord of control (Non-Tg), hSOD1^G93A^ transgenic (Tg), and Tg mice treated with GSS (Tg + GSS). GAPDH was used as a loading control. (b) Quantitative analysis of the normalized levels of TLR4, CD14, and COX2 over GAPDH. The data shown are the means ± SEM (*n* = 4) and were evaluated by one-way ANOVA and Newman-Keuls' *post hoc* test. ^∗^*p* < 0.05 and ^∗∗∗^*p* < 0.001.

It is known that GSS ameliorates systemic circulation and energy production [21]. In order to determine the effects of GSS administration in the innate immune responses of ALS mice, we examined the expression of immune system-related proteins, like TLR4, CD14, and COX2. As shown in Figure 2, the expression of TLR4, CD14, and COX2 dramatically increased by 2.4-, 1.8-, and 4.6-fold, respectively, in Tg versus Non-Tg mice. In contrast, GSS administration significantly reduced the expression of these three proteins by 1.8-, 1.5-, and 1.5-fold, respectively, in Tg mice compared with untreated Tg mice.

### 3.3. GSS Decreases Oxidative Stress-Related Proteins in the Spinal Cord of hSOD1^G93A^ Tg Mice

**Figure 3:**
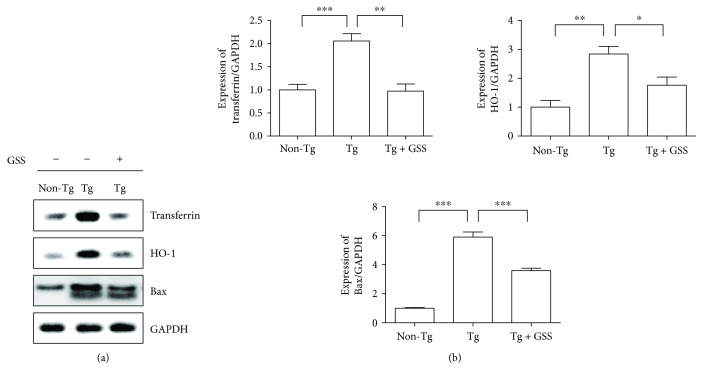
GSS treatment reduces oxidative stress in the spinal cord of hSOD1^G93A^ transgenic (Tg) mice. (a) Representative images showing the expression levels of transferrin, HO-1, and Bax in the spinal cord of control (Non-Tg), hSOD1^G93A^ Tg, and GSS-treated Tg mice (Tg + GSS). GAPDH was used to control for protein loading. (b) Quantification of the expression of transferrin, HO-1, and Bax. Data were normalized to GAPDH and represent the means ± SEM (*n* = 4). Statistical assessment was performed by one-way ANOVA and Newman-Keuls' *post hoc* test. ^∗^*p* < 0.05, ^∗∗^*p* < 0.01, and ^∗∗∗^*p* < 0.001.

Oxidative stress plays a key role in motor neuron injury and consists of a therapeutic target in ALS [31]. To evaluate the effects of GSS administration on oxidative stress in the spinal cord of hSOD1^G93A^ Tg mice, we examined the expression of transferrin, as a measure of free iron, and HO-1. As shown in Figure 3, transferrin expression was reduced by 2.1-fold in the spinal cord of hSOD1^G93A^ Tg mice after GSS administration. The expression of HO-1 was also significantly decreased by 1.6-fold. Kirkland et al. demonstrated that Bax induces oxidative stress, which is critical for cytochrome C release during programmed neuronal cell death [32]. To demonstrate the effect of GSS on cell death, we investigated the expression of Bax in the spinal cord of hSOD1^G93A^ Tg mice. We found that Bax levels significantly decreased by 1.6-fold after GSS treatment (Figure 3).

### 3.4. GSS Administration Regulates Energy Metabolism in the Spinal Cord of hSOD1^G93A^ Tg Mice

**Figure 4:**
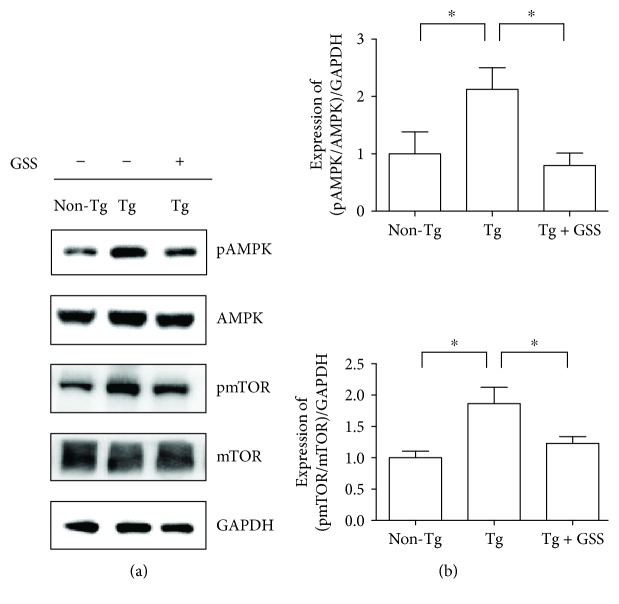
GSS modulates energy metabolism-related proteins in the spinal cord of hSOD1^G93A^ transgenic (Tg) mice. (a) Representative Western blots showing the expression of pAMPK, AMPK, pmTOR, and mTOR in the spinal cord of control (Non-Tg), hSOD1^G93A^ Tg, and GSS-treated Tg mice (Tg + GSS) mice. GAPDH was used to control for protein loading. (b) Quantification of the ratio of pAMPK/total AMPK and pmTOR/mTOR normalized to GAPDH. Data represent the means ± SEM (*n* = 4) and were evaluated by one-way ANOVA and Newman-Keuls' *post hoc* test. ^∗^*p* < 0.05.

Perera and Turner reported that metabolic homeostasis is important in ALS, suggesting that regulatory factors of energy metabolism can be candidates in the development of a drug for ALS treatment [33]. To investigate the effects of GSS on spinal cord energy metabolism in hSOD1^G93A^ Tg mice, we examined the expression of metabolism-related proteins, including pAMPK and pmTOR. We found that the levels of pAMPK were significantly lower (2.6-fold), whereas the levels of pmTOR were 1.5-fold lower in the spinal cord of GSS-treated than of untreated Tg mice (Figure 4).

## 4. Discussion

This study demonstrated that the administration of GSS inhibits neuronal cell death and neuroinflammation, as well as oxidative stress, in the spinal cord of hSOD1^G93A^ Tg mice. In addition, it reduces the immune response and regulates the defective energy metabolism, observed in these Tg mice.

ALS is an adult-onset neurodegenerative disease that specifically affects motor neurons, thus causing cell death. Approximately 5–10% of patients are diagnosed with familial ALS, caused by mutations in various genes, including SOD1, fused in sarcoma, TAR DNA-binding protein, and dynactin subunit 1; patients with sporadic ALS constitute approximately 90% of the cases, while the causes for this form of ALS have not been defined.

Although several pathological mechanisms have been identified and many therapies have been suggested for the treatment of ALS, only two drugs (riluzole and edaravone) have been approved so far by the Food and Drug Administration, albeit with no significant results regarding disease progression [20, 34]. Therefore, the development of effective treatments is essential for improving the life quality and expectancy of patients with ALS. GSS is a traditional herbal prescription comprising 12 different ingredients and has been used in Asia to treat dysmenorrhea, insomnia, and anxiety [21]. Most of its components have been shown to have anti-inflammatory effects [27].

A major characteristic of ALS pathogenesis is the neuroinflammation caused by reactive astrocytes and microglia, leading to motor neuron death. mSOD1 deletion in astrocytes and microglia of hSOD1^G93A^ mice has been shown to prolong survival [13, 35]. In addition, T lymphocytes play a neuroprotective role by modulating inflammation in hSOD1^G93A^ mice [36]. We have previously demonstrated the higher expression of Iba-1 and GFAP, as well as of proinflammatory cytokines in the spinal cord of symptomatic hSOD1^G93A^ mice [37].

Lee et al. have demonstrated that TLR4 signaling contributes to the reduction of microglia and astrocytes, thus leading to the extension of the life span of hSOD1^G93A^ mice [38]. TLR4 signaling in nonneuronal cells induces NF-*κ*B activation and triggers inflammatory mediators (such as IL-1*β*, TNF-a, and COX-2) and reactive oxygen species [39]. In addition, Zhao et al. reported that binding of mSOD1 to CD14, a co-receptor of TLR4, causes microglial activation [40]. Furthermore, inhibition of TLR4 downstream signaling via interaction with the TLR4-CD14 receptor complex prevents the neuroinflammation-induced neurodegeneration in an animal model of AD [41]. Therefore, blocking of TLR4 signaling could be helpful in reducing neuroinflammation in neurodegenerative diseases. Our experiments showed that GSS treatment attenuates neuronal death via inhibition of TLR4 signaling effectors. This suggests that GSS could be useful in preventing the neuroinflammation observed in motor neuron diseases.

Protein oxidation, nitration, carbonylation, and lipid peroxidation are suggestive of oxidative stress and have been detected in both neurons and glial cells of patients with ALS and the respective animal models. Moreover, they have been shown to correlate to disease progression [42–44]. Oxidative stress increases ROS production, thus leading to the accumulation of abnormal protein inclusions and neuroinflammatory events. Nicotinamide adenine dinucleotide phosphate-oxidase 2 (NOX2), one of the major ROS generators in the CNS, is activated in microglia and has been shown to cause neuronal toxicity in both familial and sporadic ALS [45]. In the ALS mouse model, NOX2 inhibition was reported to delay disease progression and prolong survival [46, 47]. Furthermore, ROS production is known to recruit the inflammasome, thus inducing the release of the cytokines IL-1b and IL-18 and of inflammatory and neurotoxic molecules, including COX-2 and iNOS, by microglia [48]. Moreover, oxidative stress is related to impaired iron homeostasis, involving ferritin and transferrin, thus contributing to motor neuron degeneration in ALS [49, 50]. Transferrin mediates iron internalization into cells, whereas ferritin is an intracellular iron-binding protein. Both proteins are regulated by the intracellular iron concentration, via the iron-responsive element/iron regulatory protein system. Hadzhieva et al. demonstrated that ROS increases iron concentration, as well as the expression of the transferrin receptor in the spinal cord of hSOD1^G93A^ mice [51]. In our previous study, we confirmed that transferrin expression is increased in the spinal cord of these mice [52]. In the present study, we showed that GSS treatment reduces the expression of HO-1 and transferrin in the spinal cord of symptomatic Tg mice. These data suggest that GSS treatment may alleviate the metabolic dysfunction observed in these mice.

In patients with ALS, energy metabolism is dysregulated by hypermetabolism and abnormal metabolism of lipids, which contribute to disease progression [53, 54]. In general, neurons secrete glutamate into the synaptic cleft, which is taken up by astrocytes, a process known as metabolic coupling. However, several research groups have reported the loss of glutamate transporters in spinal cord and brain astrocytes of patients with ALS, as well as excitotoxicity, induced by deficiencies in the glutamate uptake cycle [55–57]. In addition, astrocyte-neuron lactate dysregulation by the astrocytic lactate efflux transporter (monocarboxylate transporter 4, SLC16A4) results in the reduction of lactate levels in the spinal cord of hSOD1^G93A^ mice [58]. These findings imply that metabolic dysfunctions between neurons and astrocytes contribute to the energy metabolism dysfunction observed in the CNS of ALS animal models. As part of the CNS energy metabolism, the AMPK signaling pathway plays a role in the maintenance of energy homeostasis and pathophysiological states [59, 60]. Studies on ALS animal models have revealed that AMPK is activated in the spinal cord and motor neurons and causes the disease onset, consistent with the energy dysregulation and hypermetabolism seen in ALS [61–63]. In our study, GSS administration reduced the activation of AMPK and mTOR in the spinal cord of symptomatic Tg mice. These findings suggest that GSS could be used for reducing the hypermetabolism observed in ALS, by regulating AMPK-related metabolic signaling. AMPK activation is also associated with the mislocalization of human antigen R, mRNA stabilization, and cellular stress granules; however, further studies are required for investigating whether GSS could regulate RNA homeostasis.

Altogether, this study demonstrates that GSS is an effective treatment against neuroinflammation, oxidative stress, and metabolic dysfunction observed in the symptomatic ALS animal model. GSS treatment increases motor neuron survival, attenuates the activation of microglia and astrocytes, and inhibits neuroinflammation via inhibition of the TLR4 signaling pathway.

## 5. Conclusions

Since this study focused specifically on the examination of GSS effects on neuroinflammation, oxidative stress, and metabolism, further studies on motor activity, disease onset, and survival in the symptomatic ALS animal model are necessary. In addition, the bioactive compound in GSS and the molecular mechanisms of its actions need to be determined, before using this medication for the treatment of ALS patients.

## Data Availability

Data supporting the conclusions of this research are contained in the article.
